# Anti-tumor necrosis factor treatment from diagnosis is more effective and less costly than conventional “step-up” care for patients with active Crohn’s disease: a cost-effectiveness analysis from the PROFILE trial

**DOI:** 10.1093/ecco-jcc/jjaf150

**Published:** 2025-10-22

**Authors:** Nurulamin M Noor, Neil Davies, Warda Tahir, Simon Bond, Francis Dowling, Kamal V Patel, Paul A Lyons, Eoin F McKinney, Kenneth G C Smith, James C Lee, Vanessa Buchanan, Miles Parkes, PROFILE Study Group, Nurulamin M Noor, Neil Davies, Warda Tahir, Simon Bond, Francis Dowling, Kamal V Patel, Paul A Lyons, Eoin F McKinney, Kenneth G C Smith, James C Lee, Vanessa Buchanan, Miles Parkes

**Affiliations:** Department of Gastroenterology, Cambridge University Hospitals NHS Foundation Trust, Cambridge, United Kingdom; Department of Medicine, University of Cambridge School of Clinical Medicine, Cambridge, United Kingdom; Cogentia Healthcare Consulting, Cambridge, United Kingdom; Cogentia Healthcare Consulting, Cambridge, United Kingdom; Cambridge Clinical Trials Unit, Cambridge University Hospitals NHS Foundation Trust, Cambridge, United Kingdom; Cambridge Clinical Trials Unit, Cambridge University Hospitals NHS Foundation Trust, Cambridge, United Kingdom; Department of Gastroenterology, St George’s University Hospitals NHS Foundation Trust, London, United Kingdom; Department of Medicine, University of Cambridge School of Clinical Medicine, Cambridge, United Kingdom; Cambridge Institute of Therapeutic Immunology and Infectious Disease, Jeffrey Cheah Biomedical Centre, Cambridge, United Kingdom; Department of Medicine, University of Cambridge School of Clinical Medicine, Cambridge, United Kingdom; Cambridge Institute of Therapeutic Immunology and Infectious Disease, Jeffrey Cheah Biomedical Centre, Cambridge, United Kingdom; The Walter and Eliza Hall Institute of Medical Research (WEHI), Parkville, 3052, VIC, Australia; University of Melbourne, Melbourne, 3010, VIC, Australia; Genetic Mechanisms of Disease Laboratory, The Francis Crick Institute, London, United Kingdom; Department of Gastroenterology, UCL Institute of Liver and Digestive Diseases, Royal Free Hospital, London, United Kingdom; Cogentia Healthcare Consulting, Cambridge, United Kingdom; Department of Gastroenterology, Cambridge University Hospitals NHS Foundation Trust, Cambridge, United Kingdom; Department of Medicine, University of Cambridge School of Clinical Medicine, Cambridge, United Kingdom

**Keywords:** early effective therapy, top-down, -effectiveness, Crohn’s disease, new diagnosis, anti-TNF, biosimilar, intravenous, subcutaneous

## Abstract

**Background:**

The PROFILE trial demonstrated that “top-down” therapy in Crohn’s disease, with combination infliximab and immunomodulator from diagnosis, had superior efficacy and safety over 1 year compared to conventional “accelerated step-up” management. The current study aimed to evaluate the cost-effectiveness of these alternative treatment strategies.

**Methods:**

A Markov model was developed for the cost-effectiveness of “top-down” compared to “accelerated step-up” treatment. Parameters were informed by individual patient data from PROFILE and published data. Use of anti-tumor necrosis factor (TNF) therapy was modeled, using real-world contract drug costs from 18 PROFILE sites. Key model outcomes included healthcare costs (drug acquisition, drug administration, disease management, hospitalization, and surgery) and health outcomes [quality-adjusted life years (QALYs) gained] measured over a 5-year time horizon.

**Results:**

The base-case cost-effectiveness analysis indicated that a “top-down” strategy dominated over an “accelerated step-up” approach. Initiating intravenous infliximab from diagnosis yielded an incremental gain of 0.17 QALYs per patient over a 5-year period, and was less costly, saving £1681 per patient over the same time frame. Similar clinical benefits were obtained when modeling for subcutaneous infliximab and adalimumab. The greatest cost savings were with adalimumab, totaling £10 059 per patient over 5 years. Sensitivity analyses supported robustness of the results, showing “top-down” to be the most cost-effective option in 98.7% of model simulations.

**Conclusions:**

“Top-down” treatment from diagnosis with anti-TNF results in lower healthcare resource use and better clinical outcomes in patients with Crohn’s disease compared to an “accelerated step-up” strategy.

## 1. Introduction

Crohn’s disease is an inflammatory bowel disease prone to recurrent flares and typically characterized by progression to complications including intestinal strictures and fistulae.^1^^,^^2^ This chronic condition can have a substantial impact on quality of life for patients and results in considerable healthcare costs.^3^^,^^4^ The conventional approach to disease management has historically been through sequential “step-up” therapy, where treatment intensity is gradually increased until the tendency to flare is controlled.^5^ In recent years this has been challenged by evidence that earlier use of more effective therapies improves outcomes.^6–8^ We recently reported the results of the PROFILE trial, in which we demonstrated that starting patients with active Crohn’s disease at diagnosis on “top-down” therapy with combination infliximab and an immunomodulator resulted in significantly improved clinical outcomes compared to “step-up” therapy, even when the latter was applied over an accelerated timeframe.^9^

The two key concerns relating to early use of biologic therapy in Crohn’s disease have been the potential risk of adverse events, particularly infections relating to immunosuppression, and the cost of such treatment approaches. In the PROFILE trial we observed that very early biologic therapy was not associated with an increased risk of infections or serious infections, consistent with evidence from the PYRAMID registry.^10^ We also found that the proportion of patients in PROFILE experiencing serious adverse events was lower with “top-down” treatment compared to “accelerated step-up” care, notably including a 10-fold reduction in need for urgent abdominal surgery within 1 year of diagnosis.

In the pre-biologics era costs of care for the population of individuals with Crohn’s disease were dominated by need for surgery and inpatient costs.^11^ With the advent of biologic therapies this shifted. Most patients with complex Crohn’s disease could now be managed in the outpatient setting, but costs of care came to be dominated by the expense of originator biologic therapies.^12–14^ This was the case even where only a modest proportion of patients were receiving such treatments.

In 2013, the European Medicines Agency approved use of the biosimilar infliximab, based on evidence of its equivalent efficacy to the originator formulation.^15^ This was followed in 2018 by approval of biosimilar adalimumab for Crohn’s disease. The years since have seen dramatic falls in the costs of these anti-tumor necrosis factor (anti-TNF) medications by as much as 90% in some countries—enabling their use in patients with a much broader spectrum of disease severity.^16^ These price drops and increased availability have revolutionized the treatment landscape and mandated a re-evaluation of some previous health economic assumptions relating to use of early anti-TNF treatment.^17^ Having demonstrated superior clinical outcomes with “top-down” infliximab and immunomodulator combination therapy in PROFILE, we here sought to undertake a health economic analysis of this strategy in adults newly diagnosed with active Crohn’s disease based on contemporary real-world costs.

## 2. Methods

### 2.1. Study design and participants

This health economic study was a pre-planned analysis using data from the PROFILE trial. PROFILE was an open-label, multicenter, randomized controlled trial conducted in 40 hospitals in the UK.^9^ The trial included 386 adults newly diagnosed with active Crohn’s disease defined by the combination of Harvey–Bradshaw Index ≥7, elevated C-reactive protein (> upper limit of normal) or fecal calprotectin (≥200 µg/g) or both, and endoscopic evidence of active inflammation [simple endoscopic score of Crohn’s disease activity of ≥6 (≥4 if ileal-only disease)]. Patients were randomized to either “top-down” treatment with infliximab plus immunomodulator from diagnosis or an “accelerated step-up” strategy and primary results reported after 1 year follow-up.^9^^,^^18^ The current cost-effectiveness analysis modeled the follow-up period to a further 5-year time horizon (Table S1). Results are reported in accordance with the Consolidated Health Economic Evaluation Reporting Standards 2022 (CHEERS 2022) Statement (Supplementary Appendix).^19^

### 2.2. Model structure

A *de novo* regression-based Markov model was developed. The model structure defined three mutually exclusive health states for each patient (Figure S1). All patients were assumed to enter the model at the time of treatment initiation, being allocated to either “top-down” or “accelerated step-up” treatment. The key parameters considered included healthcare resource use (drug costs, infusion costs, outpatient costs, hospitalizations, and surgeries), and quality of life metrics. Costs and outcomes were measured over a 5-year time horizon and discounted at a rate of 3.5%, in accordance with National Institute for Health and Care Excellence (NICE) guidance.^20^ Discounting in the model reflects the convention that the value ascribed to costs and health benefits occurring in the future is lower than their value in the present. Given the recent availability of subcutaneous (SC) formulations of infliximab as well as adalimumab, additional scenarios were explored to consider the cost-effectiveness of using SC anti-TNF medication as part of a “top-down” treatment strategy.

### 2.3. Data

**Figure 1.**
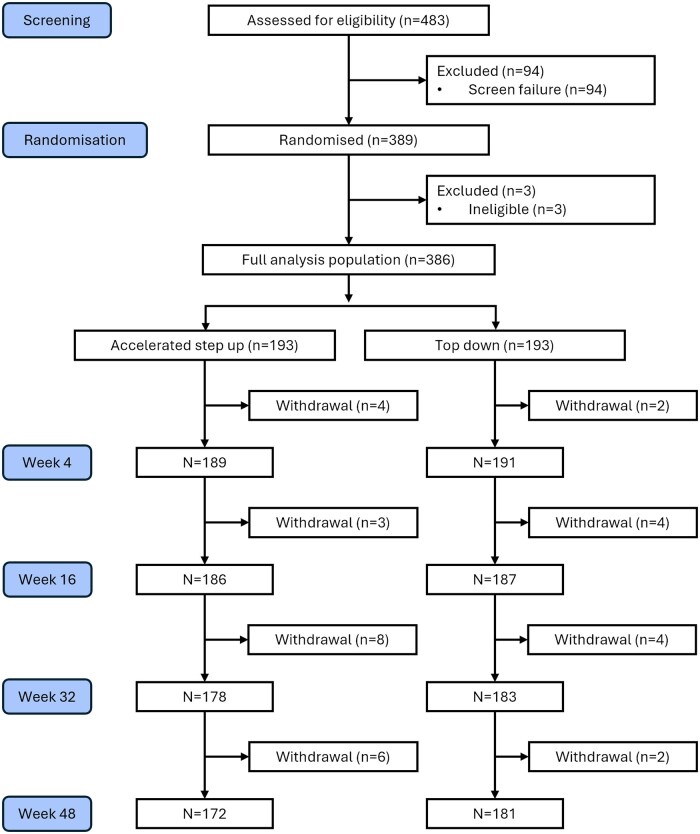
CONSORT flow diagram of PROFILE participants.

The intervention considered in the model was “top-down” treatment and the comparator was the “accelerated step-up” (conventional) treatment strategy, based on the PROFILE trial protocol.^18^ Allocation of patients in the PROFILE trial between “top-down” and “accelerated step-up” strategies is shown in Figure 1 and patient baseline characteristics are provided in Table S2. The treatments allocated to participants in each arm of the model were as follows:

- All patients in PROFILE started a course of oral steroids at enrollment.
- Patients allocated to “top-down” treatment initiated anti-TNF therapy and an immunomodulator as soon as possible, while completing their steroid taper. The PROFILE protocol stipulated that the anti-TNF therapy should be intravenous (IV) infliximab—but allowed adalimumab in certain circumstances.
  - During the later stages of the PROFILE trial, SC infliximab became widely available throughout the UK, and therefore the model also included the option of SC infliximab.
  - In the model base-case, all patients received IV infliximab induction to align with the PROFILE trial protocol.
- In the model, patients in the “top-down” arm experiencing a disease flare could receive an additional course of oral steroids and have anti-TNF dose optimization.
- Those experiencing any further flare were deemed to be non-responders to anti-TNF, as per the PROFILE trial. In the model, patients were then assumed to switch to an alternative treatment in the following proportions: ustekinumab (30%), risankizumab (30%), upadacitinib (30%), vedolizumab IV (5%), or vedolizumab SC (5%). This was based on licensed and available therapies for Crohn’s disease in the UK at the time the analysis was performed.

For patients allocated to “accelerated step-up” treatment in PROFILE, those experiencing a flare during or following the index course of steroids were treated with a more prolonged course of steroids plus an immunomodulator.

- If patients in “accelerated step-up” experienced a second flare then IV infliximab was initiated alongside the immunomodulator. In the model the final treatment step in the event of non-response or intolerance to anti-TNF medications was assumed to be an alternative “advanced therapy,” as described above for the “top-down” arm.

### 2.4. Clinical outcomes

In the model all patients started in the active disease health state and could either remain in this state, or transition to remission or (theoretically) death (Figure S1) at each monthly model cycle. Costs and health outcomes assessed through quality-adjusted life years (QALYs) were measured over a 5-year time horizon in the model base-case.

Remission was used in the model to help predict health outcomes by assigning differentiated utilities, based on a patient’s remission status. Patients who achieved and maintained remission were expected to have a higher quality of life than those not in remission. Due to lack of consensus on how long-term remission should be optimally defined in Crohn’s disease, the model included two separate definitions based on PROFILE trial outcomes. One was combined clinical and biochemical remission, defined as a combination of [Harvey–Bradshaw index (HBI) score <5] plus [C-reactive protein (CRP) <upper limit of normal (ULN) and calprotectin <200 µg/g]. This combined clinical and biochemical remission definition was used for the base-case analysis. The alternative definition focused on quality of life, measured using the inflammatory bowel disease questionnaire (IBD-Q). For this analysis, remission was defined as an IBD-Q score >170.^21^ The outcomes using the quality-of-life definition of remission were explored as a scenario analysis within the model.

During the first 48 weeks of the model the observed remission rates from the PROFILE trial were applied. After the 48-week PROFILE follow-up period, extrapolation was required to predict the proportion of patients in remission over time for each treatment approach. To analyze the temporal trend in remission rates from PROFILE, a generalized binomial mixed-effects model was fitted to the PROFILE individual patient data (IPD) to predict the outcome (remission vs non-remission/active disease) based on both fixed and random effects. Mixed effects models extend the traditional model framework by the addition of “random effect” terms. This enables the model to account for potential unobserved heterogeneity across groups. It assumes that the “random” unobserved heterogeneity is uncorrelated with independent, observed variables that are modeled, such as age, sex, or prior treatment. The generalized binomial mixed-effects model used to predict remission was represented as:


$${\mathrm{logit}(P(\mathrm{Remission}}_{ij}))=\;{\;\beta }_{0}+{\;\beta }_{1}{\mathrm{Time}}_{ij}+{\;\beta }_{2}{\mathrm{Baseline}\;\mathrm{HBI}}_{ij}+{\;\beta }_{3}{\mathrm{Top}\;\mathrm{down}\;\mathrm{strategy}}_{ij}+\omega_{i}+\;\epsilon_{ij}$$

where ${\mathrm{Remission}}_{ij}$ denotes the binary outcome repeatedly collected for each individual i=1,…,N observations at multiple time points =1,…,J. The model parameters include an intercept $\beta_{1}$ and the coefficients ($\beta_{2},\ldots ,\beta_{P+1}$) associated with the predictors, while $\omega_{i}$ and $\epsilon_{ij}$ are two random terms: $\epsilon_{ij}$ is the standard linear error term and $\omega_{i}$ is the random intercept (ie, mixed effects) which captures the “random” variation in outcomes between individuals. Mixed models for repeated measures (MMRMs) were favored because they are able to consider the correlation between repeated observations from the same patient. Given remission was a post-randomization variable, it must be acknowledged that its use in the economic model assumes that there are no confounders that influence either the chances of remission or health-related quality of life outcomes.

A range of remission models were explored based on clinical input. The final list of covariates used were decided upon using the backward selection method, whereby only clinically relevant and statistically significant covariates were included. The final model specification applied in the base-case included the following covariates: weeks from treatment randomization, baseline HBI score, and a binary variable to identify the treatment arm.

After fitting the regression model, the fixed effects coefficients were extracted and transformed into predicted probabilities using a logistic function:


$$\mathrm{Probability}=\frac{1}{1+\text{exp}(-\mathrm{logit}(P(\mathrm{Remission})))}$$

This allowed for practical prediction of the likelihood of remission beyond the PROFILE trial primary follow-up period of 48 weeks. For the “top-down” strategy, treatment discontinuation rates from anti-TNF were modeled by fitting an exponential (constant risk) parametric function to the PROFILE time on treatment data. For the “accelerated step-up” arm, as anti-TNF was not the first treatment step in the pathway, a low number of discontinuation events over the PROFILE follow-up period precluded fitting parametric functions. Treatment discontinuation rates based on loss of response to anti-TNF were therefore modeled using data from the PANTS long-term extension study (Figure S2).^22^ Although the use of external data breaks randomization, this was potentially a conservative approach as the PANTS data predict a higher discontinuation rate from anti-TNF than the few events observed during PROFILE.

To investigate the long-term benefits of a “top-down” treatment strategy versus the “accelerated step-up” strategy, the model assumed convergence in the treatment effect relating to remission. In the base-case, the model assumed the reduced benefits associated with the “accelerated step-up” strategy would eventually normalize to that of the “top-down” group, given the increasing rate of anti-TNF use over time in this group. Therefore, in the model, remission rates in both arms start to converge after 2 years in a linear trajectory. After a further 6 months, the remission rates in the two arms were assumed to fully align, and no additional benefits from the “top-down” strategy, with regard to remission rates, were included in the model (Table S3).

A Poisson regression model was used to examine the difference in number of hospitalizations, surgeries, and flares between “top-down” and “accelerated step-up” patients in PROFILE. The analysis indicated that patients in the “accelerated step-up” arm were 5.6 times more likely to require surgery, 13.5 times more likely to experience a flare requiring treatment escalation, and 1.6 times more likely to be admitted to hospital, and when patients were admitted they stayed on average 1.7 times longer in hospital. Similar to the long-term impact on remission, the model assumes the benefits seen from the reduced need for hospitalization, surgeries, and number of flares in the “top-down” arm will not be indefinite. As such, the model assumes that the rate at which patients experience hospitalization, surgery, and flares starts to converge in the “step-up” arm to the rates seen in the “top-down” arm after 3 years. By 3.5 years the model conservatively assumes the rates of hospitalization, surgery, and flares are the same across the two treatment arms until the end of the model time horizon.

### 2.5. Costs of medication

The monthly acquisition and administration costs associated with each treatment were weighted by the proportion of patients receiving each treatment. All drug acquisition costs were sourced from the drugs and pharmaceutical electronic market information tool (eMIT) or British National Formulary (BNF) if not available from eMIT. Where there were multiple branded options available, the lowest cost was selected. In the base-case, the model assumes 100% wastage (ie, no sharing of vials across patients). However, the impact of this assumption is explored in scenario analyses. In the base-case, the model also assumed all infliximab usage is IV. The dosing assumptions were as per the product’s summary of medical product characteristics (SmPC).

Real-world contract costs for IV infliximab, SC infliximab, and SC adalimumab were obtained from anonymized responses received from PROFILE trial investigators (18 responses obtained from 40 sites) in August 2024. This was similar to the real-world costing procedure used by the TISKids health economic analysis, where four responses were received from 10 sites.^23^ In the model base-case, an average discount of 84.50% was applied to the infliximab list price based on aggregated data extracted from questionnaire responses regarding current “real-world” infliximab costs from 18 PROFILE trial centers (Table S4). The unit cost associated with administration of IV infliximab was £474.94 per administration. This was sourced from the national schedule of NHS costs (NHS reference costs) and a UK stakeholder survey of infusion centers to inform average UK costs associated with the administration of IV and SC infliximab in patients with rheumatoid arthritis and IBD.^24^^,^^25^ Since the SmPC for IV infliximab indicates that response should usually be achieved within 12 weeks of treatment, the model accounted for a proportion of IV infliximab patients requiring an escalated maintenance dose. The proportion of patients to which the escalated dose was applied was determined based on the proportion of patients who experienced a flare within the first 16 weeks (nearest trial time-point to 12 weeks) of treatment in the PROFILE trial “top-down” arm. In the base-case, the model therefore assumed 8.29% (16 out of 193) of patients required an escalated maintenance dose of infliximab IV. Ongoing dose escalation in the maintenance phase was factored in for the full 5-year model time horizon and higher levels of dose escalation were also explored as a scenario analysis.

With regard to immunomodulator usage, modeling was based on data obtained for the three options provided in the PROFILE trial protocol—and assumed that patients received either azathioprine (82.35%), low-dose mercaptopurine and allopurinol (7.38%), or methotrexate and folic acid (10.27%). The acquisition costs for these treatments are summarized in Table S5. Oral treatments were assumed to incur no administration costs. Subcutaneous treatment with methotrexate was assumed to be self-administered, incurring a one-off cost of a 30-min nurse visit (£26.50) for training on how to self-administer.^26^ Significant healthcare utilization costs such as hospitalization relating to adverse events and/or intolerance to immunomodulators were accounted for in the model and based on PROFILE 1-year primary follow-up data. Further decreased rates of immunomodulator use were factored in for each year of the model (Figure S2). Disutilities from any severe adverse events were also included.

The model assumed that for disease flares, a course of steroids was initiated. The costs of steroid medication were applied as a one-off cost within the model, at the time of disease flare, with negligible costs of steroid medication based on UK prescribing costs. Healthcare resource use such as staff time and consultations and hospital admissions resulting from disease flares were already captured within the health resource use costs of the model, which were informed by PROFILE. Additional out-patient resource use, such as endoscopies, laboratory results, and radiological investigations, were not explicitly captured in the model, due to the protocolized nature and high intensity of investigations in the PROFILE trial impacting the generalizability of such data to standard clinical practice. In excluding these investigations, the model was conservative as it effectively assumed that the number of endoscopies, laboratory results, and radiological investigations undertaken would not differ between the two treatment strategies. However, in clinical practice, we might expect the number of such investigations required in the “accelerated step-up” arm would be higher than the “top-down” arm, due to the higher number of flares in the former group as seen in PROFILE. This uncertainty was accounted for in a scenario analysis, based on resource use by remission status in PROFILE.

As described above, subsequent treatment in the event of infliximab non-response or loss of response was assumed to comprise ustekinumab, upadacitinib, risankizumab, or vedolizumab (IV or SC). The acquisition costs of these treatments are reported in Table S6. The total annual acquisition costs associated with subsequent treatment following anti-TNF are reported in Table S7.

### 2.6. Health resource use costs

Healthcare resource use (HRU) such as outpatient visits, emergency department attendance, and therapist appointments were measured throughout PROFILE at weeks 4, 16, 32, and 48. Statistical analyses were conducted to examine whether HRU differs by treatment arm, adjusting for differences in follow-up time of patients in the PROFILE trial.

A Poisson regression model with a covariate to adjust for time was applied to examine the differences in HRU frequencies between patients on “top-down” and “accelerated step-up” therapy. Unit costs were then applied to the HRU frequencies (Table S8). These unit costs were based on NHS reference costs, therefore incorporating costs of investigations and initiation of disease management. Due to the assumed convergence in efficacy of the treatment strategies after 2.5 years in the base-case, the model also assumes that the HRU costs associated with the ongoing management of Crohn’s disease for each treatment strategy converge after 2.5 years.

Surgery costs were calculated as a weighted average cost of different surgery types incurred by patients and the assumed length of stay in hospital following a surgery (Table S9). Leveraging clinical expert-validated assumptions from a previous NICE technology appraisal of ustekinumab for moderate to severely active Crohn’s disease after previous treatment (TA456),^27^ the model assumed that surgery comprised 20% day cases, 10% <5-day stays and 70% >5-day stays. The costs of each surgical category, to which these proportions were applied in the model, were calculated as a weighted average of procedures in the small and large intestine, using frequencies and costs sourced from NHS reference costs. These costs are summarized in Table S10. The cost of surgery in the model was applied as a lump-sum cost incurred at the time of surgery in the model, based on the average monthly rate of surgery in each arm (Table S11).

### 2.7. Quality of life methods

Health-related quality of life (HRQoL) was measured using both IBD-Q and the EQ-5D-5L at baseline and weeks 16, 32, and 48 of PROFILE. In line with the NICE reference case, utility values were generated from the PROFILE IPD by mapping the EQ‑5D‑5L descriptive system data onto EQ‑5D-3L values.^28^ Once EQ‑5D-3L values were established, utility values were generated by remission status in order to assign health state utility values for those experiencing active disease versus remission by fitting an MMRM to the EQ-5D-5L utility values. The final model specification applied in the base-case includes the following baseline/time invariant covariates: baseline EQ-5D, baseline HBI score, ulcer sub-score at baseline, and steroid usage within the 3 months prior to PROFILE enrollment. On/off biologic status, remission status, and an interaction term between biologic use and remission status were included as time-variant covariates. The model assumes that for patients undergoing surgery, their health state utility is equivalent to the lowest health state utility value, which is the non-remission health state utility value for those who are not on biologics, and assumed to last 1 month. This approach is in line with previous methodology applied in NICE TA456.^29^ A disutility of 0.05 was applied to patients experiencing a flare to account for the short-term reduction in quality of life as a result of the flare.^30–32^ The disutility is applied in the model as a one-off disutility at the time a flare occurs and implicitly assumes the worsening of QoL due to a flare lasts 1 month.

### 2.8. Base-case analysis and sensitivity analyses

Data were analyzed based on the treatment strategy (“top-down” or “accelerated step-up”) that was initiated. To assess the robustness of the results under a variety of different assumptions, we performed three separate sensitivity analyses, namely scenario analysis, one-way sensitivity analysis (OWSA), and probabilistic sensitivity analysis (PSA) (Supplementary Appendix).

### 2.9. Validation

A comprehensive model validation was performed in which the internal validity, face validity, and external validity of the model were assessed. Several internal quality control procedures were undertaken to verify the results of the de novo cost-effectiveness model. All source inputs and calculations in the Excel model were generated by one researcher and verified by another independent researcher to ensure accuracy. Quality control also included a line-by-line audit of the Visual Basic for Applications (VBA) code used in the model. In addition, the model structure, setting, assumptions, input, and data were reviewed by health economists who have experience in model construction. Face validity was assessed by comparing the model’s predicted remission output against expert clinical opinion. External validity was evaluated by comparing the model results against previous cost-effectiveness analyses in Crohn’s disease. There is a paucity of cost-effectiveness data in adult Crohn’s disease, particularly around use of anti-TNF from diagnosis. Therefore, literature from pediatric Crohn’s disease and from adult Crohn’s disease patients receiving induction-only anti-TNF treatment were assessed.^23^^,^^32^

## 3. Results

For patients with newly diagnosed Crohn’s disease, a “top-down” treatment strategy using anti-TNF biosimilar medication was less costly and more effective than an “accelerated step-up” strategy over a 5-year time horizon (Table 1). “Top-down” treatment was associated with lower total costs and higher QALYs for the base-case analysis using IV infliximab. This was also true of the modeled analyses using SC infliximab and SC adalimumab.

**Table 1.** Costs and QALYs of “top-down” versus “accelerated step-up” over a 5-year time horizon.

Treatment strategy	Total healthcare costs (£)	Total QALYs	Cost difference	QALYs difference	ICER (£/QALY)
Top-down	£28 399	3.5982	–	–	–
Accelerated step-up	£30 079	3.4258	£1681 higher cost	0.1724 lower QALY	Top-down dominates

ICER, incremental cost-effectiveness ratio; QALY, quality-adjusted life years.

### 3.1. Costs

The “top-down” strategy was less costly than “accelerated step-up” treatment across all categories of expenditure apart from drug administration (Table S11**)**. The largest cost difference between the arms was observed in hospitalizations and abdominal surgeries, where the costs observed in the “accelerated step-up” arm were more than double those seen in the “top-down” arm (Table S11). As anticipated, the majority of acquisition costs observed in the “top-down” arm related to anti-TNF treatment and its administration (Table S12), whereas in the “accelerated step-up” arm they related to the range of biologic treatments projected in the model to be needed after anti-TNF failure. Ultimately, despite the higher initial drug administration costs for patients treated with the “top-down” strategy, total healthcare expenditure in this arm was lower than “accelerated step-up” treatment by £1681 per patient for IV infliximab.

### 3.2. Effectiveness

Over a 5-year time horizon, patients treated with a “top-down” strategy were less likely to experience flares, require hospitalization, or undergo surgery, and required fewer hospital days as inpatients. The more favorable outcomes achieved with “top-down” treatment led to greater HRQoL, and resulting QALYs, in patients following this treatment strategy compared to those who received “accelerated step-up” treatment (Table 1 and Table S13). The mean number of flares per patient in the “accelerated step-up” arm was 5.0 compared to 0.6 in the “top-down” arm. The number of urgent, extra “ad hoc” hospital outpatient clinic visits projected across the 5 years was lower for “top-down” compared to the “accelerated step-up” arm (1.7 vs 3.4 extra clinics per patient; Table S14). Likewise the length of stay in hospital was lower for patients in the “top-down” arm compared to those in the “accelerated step-up” group (2.0 vs 4.7 days hospitalized per patient; Table S14). According to the model, patients in the “accelerated step-up” group experienced 3.9 times more surgeries over 5 years than those in the “top-down” group. The model effectiveness results take into account the assumed convergence in clinical outcome rates between the two treatment groups 3.5 years after diagnosis (Table S14).

### 3.3. Cost-effectiveness

The outcome of a cost-effectiveness model is typically presented in the form of an incremental cost-effectiveness ratio (ICER). An ICER depicts the additional or “incremental” cost associated with each additional (incremental) unit of health (QALY) achieved with an intervention over the modeled time horizon. In the present study, “top-down” treatment was both less costly and clinically more effective than “accelerated step-up” treatment (Table S15). Given that “top-down” was thus a dominant treatment strategy, calculation of an ICER was not necessary.

### 3.4. Sensitivity analyses

Alternative scenarios were explored to examine differences in prescribing (ie, alternative anti-TNF drugs), modeling assumptions and data sources underpinning the model. “Top-down” remained a dominant treatment strategy when use of alternative anti-TNF drugs (SC infliximab or adalimumab) was modeled. Similar clinical benefits to IV infliximab were obtained when modeling use of “top-down” SC infliximab or adalimumab, but greater cost savings were seen with adalimumab, totaling £10 059 per patient over 5 years compared to “accelerated step-up” treatment.

Of the 23 scenarios analyzed (Table S16), only two resulted in “top-down” not being considered the more cost-effective treatment strategy at the upper end of NICE’s willingness to pay (WTP) threshold of £30 000/QALY. These scenarios (3 and 5) related to the use of undiscounted anti-TNF “list” prices for IV and SC infliximab (nominal nationally set tariffs without any real-world discount applied). These undiscounted prices were considerably higher than the real-world “contract” prices used in the base-case, which were an average of the cost of medication being paid across 18 PROFILE sites.

**Figure 2.**
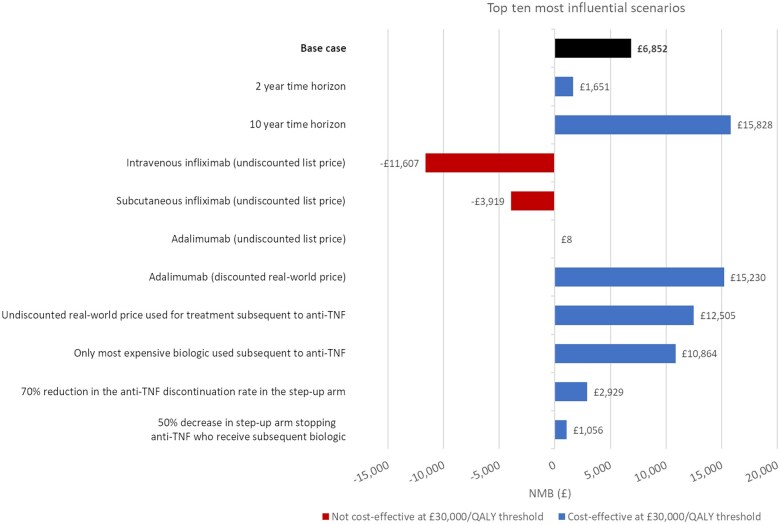
Top 10 most influential scenarios on cost-effectiveness. The base-case analysis (black bar) was over a 5-year time horizon with cost-effective scenarios (blue bars) and non-cost-effective scenarios (red bars) presented. Undiscounted list price=nominal nationally set tariff without real-world discount applied. Discounted real-world price=average cost of medication paid across 18 PROFILE sites. Different utility sources included: NICE TA456 appraisal for ustekinumab in Crohn’s disease,^27^ and infliximab pricing in international economic evaluations in inflammatory bowel disease.^49^ TNF, tumor necrosis factor; QALY, quality-adjusted life years.

An OWSA was undertaken to determine the impact of independently varying key model assumptions (Figure 2). The most sensitive parameters in the OWSA were the costs associated with IV infusions, the anti-TNF discontinuation rate in the “accelerated step-up” arm, and the coefficients that dictated the proportion of patients on anti-TNF in both arms.

**Figure 3.**
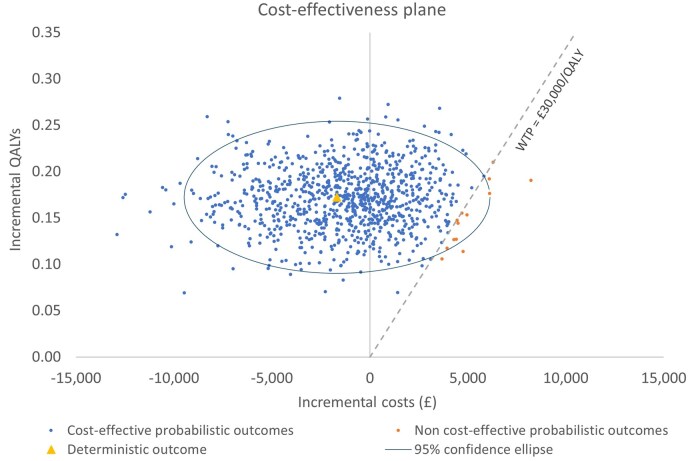
Probabilistic sensitivity analysis on the cost-effectiveness plane, showing cost-effectiveness of “top-down” versus “accelerated step-up.” These findings show cost-effectiveness based on a WTP threshold of £30 000/QALY, which is typically used by organizations such as NICE. Over 1000 simulations, “top-down” was the most cost-effective option in 98.7% of model simulations. QALY, quality-adjusted life years; WTP, willingness to pay.

A PSA was undertaken to explore the uncertainty around key model parameters. The PSA was conducted by varying model parameters using their upper and lower bound values and an assigned distribution for the values within these bounds. In total, 1000 simulations were run for the PSA, by which time the ICER had converged to a stable mean. The probabilistic results were in line with the base-case results (Table S16**)**, with “top-down” treatment remaining dominant over “accelerated step-up” care. The PSA iterations presented as scatter points on the cost-effectiveness plane (Figure 3) also showed that ­“top-down” treatment was the dominant strategy. Indeed, “top-down” therapy was the most cost-effective option in 98.7% of model simulations.

The “top-down” treatment strategy was associated with both lower costs and higher QALYs than “accelerated step-up” care (Figure S3A and B). The cost-effectiveness acceptability curve (Figure S4) shows that the probability of “top-down” treatment being cost-effective rose in line with increasing WTP thresholds, whereas the probability of “accelerated step-up” treatment being the most cost-effective strategy decreased as the WTP threshold increased.

## 4. Discussion

This health economic analysis demonstrates that “top-down” treatment with anti-TNF therapy from time of diagnosis of Crohn’s disease results in lower overall healthcare costs compared to an “accelerated step-up” strategy. This finding sits alongside the improved clinical outcomes demonstrated here (longer periods in remission, fewer disease-related flares, and enhanced quality of life) and in the primary report of the PROFILE trial,^9^ and means that in health economic terms the “top-down” strategy dominates “accelerated step-up” approaches.

The efficacy of anti-TNF therapy in Crohn’s disease, particularly when combined with an immunomodulator, has long been recognized and was reinforced in the PROFILE trial.^9^^,^^33^ Historically, however, there was a view that the immunosuppression produced by these therapies might increase the risk of adverse events, particularly infections, and that in the pre-biosimilar era it would anyway be unaffordable to prescribe biologic therapies widely. With regard to the former point, the PROFILE trial demonstrated that a “top-down” strategy actually resulted in fewer adverse events and numerically fewer serious infections compared to “accelerated step-up,” perhaps due to a combination of more effective inflammatory disease control and needing fewer steroid courses to manage flares.

One of the most striking results seen in PROFILE was the 10-fold reduction in need for urgent abdominal surgeries with use of “top-down” therapy. As well as better disease control and improved quality of life achieved by this strategy, in the context of a cost–benefit analysis it is important to recall that surgery has historically been a big driver of costs in Crohn’s disease.

Another key driver of costs in Crohn’s disease is hospitalizations. In the PROFILE primary 1-year results a marked reduction in the number of hospitalisations required for serious adverse events for patients in the “top-down” arm versus “accelerated step-up” care was noted.^9^ In the current cost-­effectiveness analysis, the model shows admissions to be not only fewer but also shorter for patients in the “top-down” arm, with an average length of stay of 4.2 days compared to 7.2 days. The combination of fewer and shorter hospitalizations in the “top-down” arm compared to “accelerated step-up” resulted in projected costs savings of £1786 per patient, over 5 years.

The base-case in our analysis assumed use of IV infliximab, as the protocol had dictated in the PROFILE trial. Costs of administering IV infliximab are, however, generally high—indeed these were identified as one of the most sensitive parameters in the OWSA. SC infliximab is increasingly available, and adalimumab has only ever been marketed in a SC formulation, both therefore avoiding the logistic complexities and costs of IV administration. We modeled use of both of these as alternatives to IV infliximab and were reassured to see that this did not affect the dominance of the “top-down” strategy in health economic terms. Indeed, perhaps unsurprisingly, the cost benefits for “top-down” use of adalimumab were quite substantial, saving over £10 059 per patient over 5 years compared to “accelerated step-up” management.

PROFILE was not the first study to point to the advantages of early, effective therapy—although no previous trials had recruited patients at diagnosis. Previously, a “top-down” approach using infliximab induction and then discontinuing after three doses showed promising signals of efficacy compared to conventional care in patients within 2 years of diagnosis of Crohn’s disease.^34^ However, with no maintenance biologic therapy arm, no difference was observed in the D’Haens study between the two treatment strategies over time.^34^^,^^35^ The CALM trial subsequently showed clinical benefit of using “early” adalimumab therapy in a cohort of patients approximately 1 year after diagnosis with a subsequent tight control monitoring strategy.^8^ This approach was demonstrated to be cost-effective.^36^

Despite growing evidence for the benefits of earlier use of advanced therapies,^37^ “step-up” approaches to care have remained widespread. Recent data from the USA demonstrated repeated corticosteroid use in a cohort of 2594 patients with Crohn’s disease.^38^ Two years after diagnosis, only 14% of patients had been started on an “advanced” therapy, and they had received a median of four courses of corticosteroids prior to initiation of this. Indeed, this ongoing use of “step-up” care and the inadequate control of active disease resulting in progression to complications^39^ has been highlighted as one of the main drivers of high healthcare costs in Crohn’s disease.^40^

It is worth noting that the European Crohn’s and Colitis Organisation (ECCO) guidelines now recommend use of early, effective therapies in patients diagnosed with active Crohn’s disease.^41^ However, many countries require supportive cost-effectiveness data before they will approve “top-down” treatment as the standard of care.

The cost-effectiveness findings from the current study are in line with the TISKids trial of early infliximab therapy in children newly diagnosed with Crohn’s disease.^42^ TISKids was a randomized controlled trial with 89 patients, and showed the clinical superiority of a “top-down” approach using infliximab versus a conventional “step-up” approach in the pediatric setting. Health economic analysis from TISKids demonstrated that “top-down” treatment was more cost-effective than conventional “step-up” management.^23^ Indeed, as in our study, “top-down” treatment dominated “step-up” strategies—being both clinically more effective and less costly. Alongside this, recent evidence from pediatric Crohn’s disease patients suggests that early disease control indeed prevents progression to more severe phenotypes and complications such as fistulizing disease.^43^

There are a number of limitations to consider when interpreting the results of our analysis. First, the model is sensitive to assumptions made, including the proportion of patients losing response to anti-TNF therapy past the observed trial follow-up period. Different scenarios were modeled to address this. They showed that even with a 70% reduction in anti-TNF discontinuation rates in the “accelerated step-up” arm, use of “top-down” anti-TNF treatment from diagnosis remained cost effective. This was true even at the lowest WTP threshold used by NICE. While these sensitivity analyses are reassuring, truly robust long-term cost–benefit analysis of the different treatment strategies based on real-world observational data will need to await the ongoing 5-year follow-up of PROFILE participants. Second, costs of medication can be highly variable. Contract pricing for medications was not evaluated from healthcare providers outside of PROFILE trial sites. However, given that sites taking part in PROFILE encompassed a broad range of hospitals and regions, the medication costs ascertained are broadly representative of “real-world” costs across the UK. Third, outpatient visits included in the model reflected only the extra “ad-hoc” visits recorded in PROFILE. This may underestimate the true number of extra contacts and outpatient visits required for patients who are being managed by “step-up” care in the real world, where the number of scheduled visits is fewer and monitoring/treatment escalation is often less timely than was the case in the PROFILE trial setting. Fourth, healthcare resource use was calculated based on data collected prospectively within PROFILE, but HRU may not have been fully reported, for example where patients forgot to record contacts or visits with healthcare providers. Furthermore, indirect costs such as reduced work productivity or unemployment^44–46^ were not captured. Total costs may therefore have been underestimated in the model. Given the randomization in the PROFILE trial, we believe that any underestimation would be balanced between groups or perhaps higher for participants on “accelerated step-up” therapy. Our modeling demonstrates that “top-down” use of adalimumab may be the most cost-effective treatment strategy, but this is based on the assumption that adalimumab would be equally effective as infliximab when used from the point of diagnosis. In addition, while our data are based on a randomized control trial of anti-TNF therapy and therefore focus on cost-effectiveness of anti-TNF treatment, the choice of first-line effective therapy may be influenced by other factors such as costs of medication, contra-indications to anti-TNF, and/or presence of other immune-mediated inflammatory diseases. Moreover, there is high heterogeneity between clinicians for choice of medication to use as second-line therapy after anti-TNF. It is plausible that proportions of patients allocated to different treatments will vary between countries based on factors including: patient characteristics, preferences, cost, availability and national guidance, and/or reimbursement. Future research should seek to determine the cost-effectiveness of different treatment sequencing strategies. Of note, inclusion criteria for the PROFILE trial required evidence of active inflammation in terms of biomarkers (raised CRP or raised calprotectin or both) and endoscopic inflammation.^18^ While most patients newly diagnosed with Crohn’s disease manifest these features, some with very mild disease do not. The results of this health economic analysis cannot be applied to patients in the latter group.

There are also several strengths of this work. This is the first study, to our knowledge, to assess the cost-effectiveness of using anti-TNF medication from diagnosis and with continued maintenance therapy in adult patients with active Crohn’s disease. One study had previously assessed cost-effectiveness of three doses of early infliximab, but with no maintenance treatment.^32^ Given that PROFILE was a randomized controlled trial, the effectiveness of the alternative strategies could be thoroughly assessed with minimal risk of confounding, unlike some previous health economic analyses that have sought to assess “early” use of anti-TNF therapy in Crohn’s disease using only observational data.^12^^,^^13^ Furthermore, remission in our study was defined by a combination of clinical and biochemical measures collected longitudinally during the PROFILE trial, rather than a snapshot of endoscopic remission at one timepoint. Combining clinical assessment with objective biochemical evidence of remission is consistent with clinical practice and the STRIDE-II guidelines which support a treat-to-target and tight control approach to care.^47^ Many health economic models are based on source data from non-representative and non-diverse populations.^48^ In this regard, PROFILE had a diverse and representative population in terms of ethnic background, highlighting the generalizability of our results. This is also the first study, to our knowledge, to demonstrate dominance of a “top-down” strategy in adults using IV infliximab, SC infliximab, and SC adalimumab. The sensitivity analyses are consistent and demonstrate robustness of the data supporting use of anti-TNF biosimilar medication for patients newly diagnosed with active Crohn’s disease.

## 5. Conclusions

Using data from the PROFILE trial, our health economic analysis demonstrates that a “top-down” strategy of IV infliximab therapy with an immunomodulator from diagnosis results in lower direct healthcare costs and more favorable clinical outcomes. “Top-down” is thus the dominant strategy over “accelerated step-up” treatment over a projected 5-year time horizon. The cost-effectiveness analysis also demonstrates that a “top-down” approach using either SC infliximab or SC adalimumab would also be a dominant strategy over “accelerated step-up” management. Given these findings, initiation of early effective “top-down” treatment should be considered the standard of care for patients newly diagnosed with active Crohn’s disease.

## Supplementary Material

jjaf150_Supplementary_Data

## Data Availability

Access to the study data and other materials is managed by the Cambridge University Hospitals R&D Study Review Committee. Please contact the corresponding author.
