# Supplementary material for: Anti-tumor necrosis factor treatment from diagnosis is more effective and less costly than conventional “step-up” care for patients with active Crohn’s disease: a cost-effectiveness analysis from the PROFILE trial
Source: J Crohns Colitis. 2025 Oct 22;19(9):jjaf150. doi: 10.1093/ecco-jcc/jjaf150 (PMC12543358; doi:10.1093/ecco-jcc/jjaf150)
Supplement: jjaf150_Supplementary_Data [file jjaf150_supplementary_data.docx]

**Supplementary appendix**

**Sensitivity analysis top-down versus accelerated step-up.**

**One-way sensitivity analysis**

A one-way sensitivity analysis (OWSA) was undertaken to determine the impact of independently varying key model assumptions. OWSA is a method for assessing how much the result of the economic model is influenced by any one parameter. To accomplish this, each variable is altered individually while keeping the others constant and the impact on the model output measured. In cases where the intervention (top-down) dominates the comparator (step-up), an ICER was not calculated. Net monetary benefit (NMB) was calculated. NMB is a summary statistic that represents the value of an intervention in monetary terms when a willingness to pay threshold for a unit of benefit (for example, the conventional standard of £20,000 to £30,000 per QALY) is known. The use of NMB scales both health outcomes and use of resources to costs, with the result that comparisons without the use of ratios (such as in ICERs) can be made. NMB was calculated as (incremental benefit x threshold) – incremental cost. Incremental NMB measures the difference in NMB between alternative interventions, a positive incremental NMB indicating that the intervention is cost-effective compared with the alternative at the given willingness-to-pay threshold.

**Probabilistic sensitivity analysis**

A PSA was conducted to explore the uncertainty around key model parameters. PSA was conducted by varying model parameters using their upper and lower bound values and an assigned distribution for the values within these bounds. 1,000 simulations were run for the PSA, by which time the ICERs had converged to a stable mean, represented by the probabilistic outcomes.

**Scenario analysis**

The model also includes functionality to conduct scenario analyses, in which model results are generated for alternative sets of assumptions. The sensitivity of the model results to changes in key assumptions was examined.

**Supplementary Table 1. Features for the base-case economic analysis.**

| **Factor** | **Value/Source** |
| --- | --- |
| Time horizon | 5 years |
| Cycle length | Monthly |
| Discount rate (costs and health outcomes) | 3.5% |
| Source of utilities | PROFILE IPD analysis |
| Source of costs and HCRU | PROFILE IPD analysis,  BNF,  eMIT,  PSSRU,  NHS national cost collection |

|  | **Top-down** | **Accelerated Step-up** |
| --- | --- | --- |
| Monthly discontinuation rate for anti-TNF | N/A* | 0.0169 |
| Annual surgery rate | 0.0119 | 0.0667 |
| Annual hospitalisation rate | 0.0953 | 0.1505 |
| Annual flare rate | 0.1126 | 1.5241 |

*In the PROFILE trial, patients in the “top-down” arm started on anti-TNF. As a result the regression coefficient from the exponential model used to estimate time on anti-TNF, inherently reflects any treatment discontinuation that occurred in “top-down” during PROFILE. Therefore a separate discontinuation rate is not needed for the “top-down” arm in this model.

**Supplementary Table 2. Baseline characteristics of PROFILE participants.**

| **Variable** | **Accelerated Step up (n=193)** | **Top down (n=193)** |
| --- | --- | --- |
| Mean age (years) | **34.0 (13.3)** | **33.3 (13.2)** |
| Sex |  | |
| Female | **88/193 (46%)** | **91/193 (47%)** |
| Ethnicity |  | |
| White | 168/193 (87%) | 171/192 (89%) |
| Other | 25/193 (13%) | 21/192 (11%) |
| Current smoker | 42/193 (22%) | 49/191 (26%) |
| Mean weight (SD; kg) | **74.9 (17.5)** | **74.7 (19.3)** |
| Disease location |  | |
| Ileal | 63/193 (33%) | 65/192 (34%) |
| Colonic | 50/193 (26%) | 53/192 (28%) |
| Ileocolonic | 80/193 (41%) | 74/192 (39%) |
| Disease behaviour |  | |
| Inflammatory (B1) | 161/190 (85%) | 169/192 (88%) |
| Non-inflammatory | 29/190 (15%) | 23/192 (12%) |
| Mean HBI score (SD) | **9.8 (2.9)** | **10.0 (2.9)** |
| Mean CRP (mg/L; SD) | 21 (26) | 19 (27) |
| Median CRP (mg/L; IQR) | 12 (5 – 24.2) | 11 (5 - 20) |
| Mean Calprotectin (ug/g; SD) | 993 (797) | 1035 (991) |
| Median Calprotectin (ug/g; IQR) | 835 (322 - >1800) | 747 (381 - >1800) |
| Mean SES-CD (SD) | 10.4 (6.0) | 10.9 (6.6) |
| Median SES-CD (IQR) | 9 (7 - 13) | 9 (7 - 14) |
| Steroid course prior to enrolment | **40/192 (21%)** | **30/193 (16%)** |
| Mean time from diagnosis to enrolment (days; SD) | 31.2 (40.0) | 24.1 (34.4) |
| Median time from diagnosis to enrolment (days; min-max) | 14.0 (0 - 191) | 9.0 (0 - 168) |
| *Randomisation strata* | | |
| *Biomarker status* |  | |
| *IBDhi* | *97/193 (50%)* | *94/193 (49%)* |
| *IBDlo* | *96/193 (50%)* | *99/193 (51%)* |
| *Disease location* |  | |
| *Colonic* | *51/193 (26%)* | *50/193 (26%)* |
| *Other* | *142/193 (74%)* | *143/193 (74%)* |
| *Endoscopic inflammation* |  | |
| *Mild* | *14/193 (7%)* | *13/193 (7%)* |
| *Moderate* | *136/193 (70%)* | *136/193 (70%)* |
| *Severe* | *43/193 (22%)* | *44/193 (23%)* |

Baseline clinical variables included in the cost-effectiveness analysis model are highlighted in bold.

**Supplementary Table 3. Composite of clinical/biochemical remission rate by treatment strategy.**

| **Time point (months after PROFILE end of trial visit)** | **Top-down** | **Accelerated Step-up** |
| --- | --- | --- |
| 6 | 57.90% | 21.68% |
| 12 | 65.27% | 40.52% |
| 18 | 68.53% | 44.84% |
| 24 | 69.59% | 49.36% |
| 30 | 69.59% | 69.59% |
| 36 | 69.59% | 69.59% |

**Supplementary Table 4. Intravenous infliximab treatment costs.**

| **Treatment** | **Dosing regimen** | | | **Vial/tablet size (mg)** | **Unit cost** | | **Source** | | **Discount** | | **Discounted unit cost** |
| --- | --- | --- | --- | --- | --- | --- | --- | --- | --- | --- | --- |
| Infliximab | Induction: 5 mg/kg at week 0, 2, and 6  Maintenance: 5 mg/kg every 8 weeks | | | 100.00 | £377.00 | | BNF | | 84.50% | | £58.43 |
| *Induction costs* | | | | | | | | | | | |
| **Dose** | | **Dose required** | **Total dose (mg)** | | | **Cost per dose** | | **Number of administrations** | | **Total cost of induction doses** | |
| 5 mg/kg at week 0, 2, and 6 | | 5 mg/kg | 400.00 | | | £233.73 | | 3.00 | | £701.18 | |
| *Maintenance costs* | | | | | | | | | | | |
| **Dose** | **Relative usage** | | | **Dose required** | **Total dose (mg)** | | **Cost per dose** | | **Number of administrations per model cycle** | | **Cost per month** |
| Standard dose: 5 mg/kg every 8 weeks | 91.71% | | | 5 mg/kg | 400.00 | | £233.73 | | 0.54 | | £127.04 |
| Escalated dose: 10 mg/kg every 8 weeks | 8.29% | | | 10 mg/kg | 800.00 | | £467.45 | | 0.54 | | £254.07 |
| **Annual cost (year 1)** | | | | | | | | | | | **£2,076.85** |
| **Annual cost (year 2+)** | | | | | | | | | | | **£1,650.81** |

**Supplementary Table 5. Immunomodulator treatment costs.**

| **Treatment** | **Vial/tablet size (mg)** | **Relative usage of vial/tablet size** | **Unit cost** | **Number of vials/pack size** | **Cost per mg** | **Dose required** | **Total dose (mg)** | **Cost per dose** | **Administrations per model cycle** | **Cost per month** |
| --- | --- | --- | --- | --- | --- | --- | --- | --- | --- | --- |
| Azathioprine | | | | | | | | | | £2.61 |
| Relative usage | | | | |  |  |  |  |  | 82.35% |
| Azathioprine | 50 | 100.00% | £1.20 | 56 | £0.0004 | 2.5 | 200.00 | £0.09 | 30.44 | £2.61 |
| Low-dose mercaptopurine & allopurinol | | | | | | | | | | £10.56 |
| Relative usage | |  |  |  |  |  |  |  |  | 7.38% |
| Mercaptopurine | 50 | 100.00% | £8.39 | 25 | £0.01 | 0.35 | 50.00 | £0.34 | 30.44 | £10.21 |
| Allopurinol | 100 | 100.00% | £0.32 | 28 | £0.00 | 100 | 100.00 | £0.01 | 30.44 | £0.35 |
| Methotrexate & folic acid | | | | | | | | | | £78.13 |
| Relative usage | | | | |  |  |  |  |  | 10.27% |
| Methotrexate | | | | | | | | | | £78.09 |
| Methotrexate (Oral) | 2.5 | 50.00% | £1.12 | 24 | £0.02 | 25 | 25.00 | £0.47 | 4.35 | £2.03 |
| Methotrexate (SC) | 5 | 50.00% | £35.45 | 5 | £1.42 | 25 | 25.00 | £35.45 | 4.35 | £154.14 |
| Folic Acid |  |  |  |  |  |  |  |  |  | £0.04 |
| Folic Acid | 5 | 100.00% | £0.25 | 28 | £0.00 | 5 | 5.00 | £0.01 | 4.35 | £0.04 |
| Weighted monthly cost | | | | | | | | | | £10.95 |

**Supplementary Table 6. Subsequent treatment acquisition costs applied in the model.**

| **Treatment** | | **Vial/tablet size (mg)** | | | | **Unit cost** | | | **Discount** | | **Discounted unit cost** | **Number of vials/pack size** | | **Cost per mg** | | **Dose required** | | **Frequency** |
| --- | --- | --- | --- | --- | --- | --- | --- | --- | --- | --- | --- | --- | --- | --- | --- | --- | --- | --- |
| Ustekinumab | | | | | | | | | | | | | | | | | | |
|  | Stelara (induction [IV]) | 130 | | | | £2,147.00 | | | 55.00% | | £966.15 | 1 | | £7.43 | | 390 | | One-off |
|  | Stelara (maintenance [SC]) | 90 | | | | £2,147.00 | | | 55.00% | | £966.15 | 1 | | £10.74 | | 90 | | Week 8, then every 12-weeks thereafter |
| Upadacitinib | | | | | | | | | | | | | | | | | | |
|  | Rinvoq (induction) | 45 | | | | £2,087.10 | | | 55.00% | | £939.20 | 28 | | £0.75 | | 45 | | Daily for 12 weeks |
|  | Rinvoq (maintenance) | 30 | | | | £1,281.54 | | | 55.00% | | £576.69 | 28 | | £0.69 | | 30 | | Daily from week 12 |
| Risankizumab | | | | | | | | | | | | | | | | | | |
|  | Skyrizi (induction) | 600 | | | | £3,326.09 | | | 35.00% | | £2,161.96 | 1 | | £3.60 | | 600 | | Week 0, 4, and 8 |
|  | Skyrizi (maintenance) | 360 | | | | £3,326.09 | | | 35.00% | | £2,161.96 | 1 | | £6.01 | | 360 | | Week 12, and every 8-weeks thereafter |
| Vedolizumab - intravenous | | | | | | | | | | | | | | | | | | |
|  | Entyvio | 300 | | | | £2,050.00 | | | 10.00% | | £1,845.00 | 1 | | £6.15 | | 300 | | Week 0, 2, and 6, then every 8-weeks thereafter |
| Vedolizumab – subcutaneous | | | | | | | | | | | | | | | | | | |
|  | Entyvio (induction [IV]) | 300 | | | | £2,050.00 | | | 10.00% | | £1,537.50 | 1 | | £5.13 | | 300 | | Week 0, 2, and 6 |
|  | Entyvio (maintenance [SC]) | 108 | | | | £1,025.00 | | | 25.00% | | £768.75 | 2 | | £3.56 | | 108 | | Every 2-weeks thereafter |
|  |  | |  |  |  |  |  |  |  |  |  |  |  |  |  |  |  |  |

**Supplementary Table 7. Total subsequent treatment acquisition costs.**

| **Treatment** | **Relative usage** | **Year 1** | **Year 2+** |
| --- | --- | --- | --- |
| Ustekinumab | 30.00% | £7,598.85 | £4,201.03 |
| Upadacitinib | 30.00% | £8,147.35 | £7,522.75 |
| Risankizumab | 30.00% | £16,858.13 | £14,100.99 |
| Vedolizumab - intravenous | 5.00% | £14,801.18 | £12,033.68 |
| Vedolizumab - subcutaneous | 5.00% | £12,949.32 | £10,028.07 |
|  | Average annual cost | £11,168.82 | £8,850.52 |

**Supplementary Table 8. Health care resource use applied in the model.**

|  | **Resource use category** | **Unit cost** | **HRU annual frequency, top-down** | **HRU annual frequency, step-up** |
| --- | --- | --- | --- | --- |
| Outpatient | IBD consultant | £195.20 | 0.30 | 0.91 |
| A&E | A&E attendance | £311.38 | 0.11 | 0.41 |
| Other | Allied healthcare professional | £63.00 | 0.09 | 0.02 |
| Total | | | £100.55 | £306.54 |
| Average cost per model cycle (month) | | | £9.11 | £27.77 |

**Supplementary Table 9. Hospitalisations costs used in the model.**

|  | **Resource use category** | **Unit cost** | **Top-down arm – annual frequency** | **Accelerated Step-up arm – annual frequency** |
| --- | --- | --- | --- | --- |
| Hospitalisation | Inpatient admission (cost per day) | £710.78 | 0.10 | 0.15 |
|  | Length of stay – per admission (days) |  | 4.17 | 7.21 |

**Supplementary Table 10. Surgery costs applied in the model.**

| **Length of stay – relative frequency** | | **Surgery type** | **Unit cost** | **Number of treatments provided per year across the NHS** | **Average cost** | **Reference** |
| --- | --- | --- | --- | --- | --- | --- |
| Surgery day case | 20% | Major small intestine procedures | £1,168 | 555 | £1,665 | NHS Reference Costs 2022/23. Day case. Currency code FF22A-D Major Small Intestine Procedures, 19 years and over, with CC Score 0–7+ |
|  |  | Major large intestine procedures | £2,302 | 433 |  | NHS Reference Costs 2022/23. Day case. Currency code FF34A-C Major Large Intestine Procedures, 19 years and over, with CC Score 0-3+ |
| Surgery <5 days | 10% | Major small intestine procedures | £7,814 | 4,229 | £7,735 | NHS Reference Costs 2022/23. Elective inpatients. Currency code FF22A-D Major Small Intestine Procedures, 19 years and over, with CC Score 0–7+ |
|  |  | Major large intestine procedures | £7,466 | 1,236 |  | NHS Reference Costs 2022/23. Elective inpatients. Currency code FF34A-C Major Large Intestine Procedures, 19 years and over, with CC Score 0-3+ |
| Surgery >5 days | 70% | Complex major small intestine procedures | £10,272 | 4,833 | £12,265 | NHS Reference Costs 2022/23. Non elective (long-stay & short-stay). Currency code FF21A-D Very Major Small Intestine Procedures, 19 years and over, with CC Score 0–8+ |
|  |  | Complex major large intestine procedures | £17,241 | 1,936 |  | NHS Reference Costs 2022/23. Non elective (long-stay & short-stay). Currency code FF30A-D Very Complex Large Intestine Procedures with CC Score 0–9+ |
|  |  |  |  | Total | £9,692 |  |

**Supplementary Table 11. Summary discounted costs over the model time-horizon (5-years).**

| **Outcome** | **Top-down** | **Accelerated Step-up** |
| --- | --- | --- |
| Acquisition | £11,713.80 | £15,768.15 |
| Administration | £14,366.45 | £8,085.08 |
| Surgery | £524.17 | £2,095.12 |
| Hospitalisation | £1,286.57 | £3,072.33 |
| Disease management | £507.57 | £1,058.39 |
| **Total** | **£28,398.56** | **£30,079.08** |

**Supplementary Table 12. Disaggregated treatment acquisition costs.**

|  | **Top-down** | **Accelerated Step-up** |
| --- | --- | --- |
| Anti-TNF | £7,502.87 | £4,117.16 |
| Immunomodulator | £510.20 | £347.45 |
| Subsequent treatment | £3,634.20 | £11,027.34 |
| Steroids (initiation and flares) | £66.52 | £276.20 |
| **Total** | **£11,713.80** | **£15,768.15** |

**Supplementary Table 13. Summary discounted QALYs over the model time-horizon (5-years).**

| **Outcome** | **Top-down** | **Accelerated Step-up** |
| --- | --- | --- |
| Remission - on biologic | 2.5312 | 1.6675 |
| Remission - off biologic | 0.0000 | 0.3384 |
| No remission - on biologic | 1.0661 | 0.9223 |
| No remission - off biologic | 0.0000 | 0.5048 |
| Surgery | 0.0031 | 0.0122 |
| Flares | -0.0021 | -0.0194 |
| **Total** | **3.5982** | **3.4258** |

**Supplementary Table 14. Summary clinical events over the model time-horizon (5-years).**

| **Outcome** | **Top-down** | **Accelerated Step-up** |
| --- | --- | --- |
| Ad hoc outpatient clinic visit | 1.68 | 3.38 |
| Emergency department visit | 0.63 | 1.45 |
| Allied healthcare professional visit | 0.51 | 0.29 |
| Surgery | 0.06 | 0.23 |
| Hospitalisations | 0.48 | 0.65 |
| Hospital days | 1.98 | 4.71 |
| Flares | 0.56 | 4.96 |

**Supplementary Table 15. Deterministic (base-case) and probabilistic cost-effectiveness results for “top-down” versus “accelerated step-up”.**

|  | **Total** | | | **Difference** | | | **Model outcomes (top-down vs step-up)** |
| --- | --- | --- | --- | --- | --- | --- | --- |
|  | **Costs** | | **QALYs** | **Costs** | | **QALYs** | **ICER (£/QALY)** |
| **Deterministic (base-case) results** | | | | | | | |
| **Top-down** | | £28,399 | 3.5982 | |  |  |  |
| **Step-up** | | £30,079 | 3.4258 | | £1,681 higher | 0.1724  lower | Dominant |
| **Probabilistic results (95% CI)** | | | | | | | |
| **Top-down** | | £28,984 (£21,660, £37,006) | 3.5769 (2.7976, 4.2753) | |  |  |  |
| **Step-up** | | £30,284 (£23,183, £39,227) | 3.4062 (2.6583, 4.1033) | | £1,300 higher (-£7,956, £3,997) | 0.1707 lower (0.1033, 0.2349) | Dominant (Dominant, 26,291) |

**Supplementary Table 16. Scenario analysis results.**

| **Scenario number** | **Structural assumption** | **Base-case scenario** | **Other scenarios considered** | **Incremental costs** | **Incremental QALYs** | **ICER (top-down vs step-up)** |
| --- | --- | --- | --- | --- | --- | --- |
|  | Base case |  |  | -£1,681 | 0.1724 | Dominant |
| 1 | Time horizon | 5 years | 2 years | £2,391 | 0.1347 | £17,747 |
| 2 |  |  | 10 years | -£10,257 | 0.1857 | Dominant |
| 3 | Anti-TNF drug | Intravenous infliximab only (with discount) | Intravenous infliximab use only (without discount) | £16,778 | 0.1724 | £97,336 |
| 4 |  |  | Subcutaneous infliximab use only (with discount) | -£3,445 | 0.1724 | Dominant |
| 5 |  |  | Subcutaneous infliximab use only (without discount) | £9,090 | 0.1724 | £52,733 |
| 6 |  |  | Adalimumab use only (with discount) | -£10,059 | 0.1724 | Dominant |
| 7 |  |  | Adalimumab use only (without discount) | £5,163 | 0.1724 | £29,951 |
| 8 | Source of health state utility values | PROFILE IPD analysis | NICE TA456 | -£1,681 | 0.1651 | Dominant |
| 9 |  |  | Bashir et al., 2023 | -£1,681 | 0.0920 | Dominant |
| 10 | Healthcare resource use | Split by trial arm (top-down versus step-up) | Split by remission status (remission versus no remission) | -£200 | 0.1724 | Dominant |
| 11 | Definition of remission | Composite clinical/biochemical remission | Quality of life remission | -£1,681 | 0.1701 | Dominant |
| 12 | Inclusion of mortality | General population and surgery-related mortality is included | No mortality is accounted for | -£1,777 | 0.1642 | Dominant |
| 13 | % of step up patients progressing to subsequent treatment | 100% of step up patients stopping treatment receive subsequent treatment | 50% of step up patients stopping treatment receive subsequent treatment | £4,115 | 0.1724 | £23,874 |
| 14 | Treatment effect convergence | Remission: Starts at 2-years, time to full convergence = 6-month  Hospitalisation/surgery/flares: Starts at 3-years, time to full convergence = 6-months | All: Starts at 3-years, time to full convergence = 6-months | -£1,681 | 0.1843 | Dominant |
| 15 |  |  | All: Starts at 2-years, time to full convergence = 6-months | -£897 | 0.1648 | Dominant |
| 16 |  |  | All: No convergence | -£3,068 | 0.2021 | Dominant |
| 17 | Treatment wastage | Wastage included | No wastage (100% vial sharing) | -£1,908 | 0.1724 | Dominant |
| 18 | Remission | PROFILE data used up to 48-weeks then modelled rates beyond | Modelled remission data only | -£1,681 | 0.1863 | Dominant |
| 19 | Anti-TNF discontinuation rate in the step-up arm | PANTS data | Discontinuation rate is halved | £2,242 | 0.1724 | £13,005 |
| 20 | Subsequent treatment | \|  \| Relative usage \| \| --- \| --- \| \| Vedolizumab - intravenous \| 5.00% \| \| Vedolizumab - subcutaneous \| 5.00% \| \| Upadacitinib \| 30.00% \| \| Risankizumab \| 30.00% \| \| Ustekinumab \| 30.00% \| | Only least expensive biologic (ustekinumab) is used as subsequent treatment | £1,180 | 0.1724 | £6,846 |
| 21 |  | \|  \| Relative usage \| \| --- \| --- \| \| Vedolizumab - intravenous \| 5.00% \| \| Vedolizumab - subcutaneous \| 5.00% \| \| Upadacitinib \| 30.00% \| \| Risankizumab \| 30.00% \| \| Ustekinumab \| 30.00% \| | Only most expensive biologic (risankizumab) is used as subsequent treatment | -£5,693 | 0.1724 | Dominant |
| 22 |  | Discount based on clinical opinion | No discount (list prices) | -£7,333 | 0.1724 | Dominant |
| 23 | Infliximab IV dose escalation | 8.29% of patients on infliximab IV require dose escalation | 20% of patients on infliximab IV require dose escalation | -£1,320 | 0.1724 | Dominant |

**Supplementary Figure 1. Model structure.**


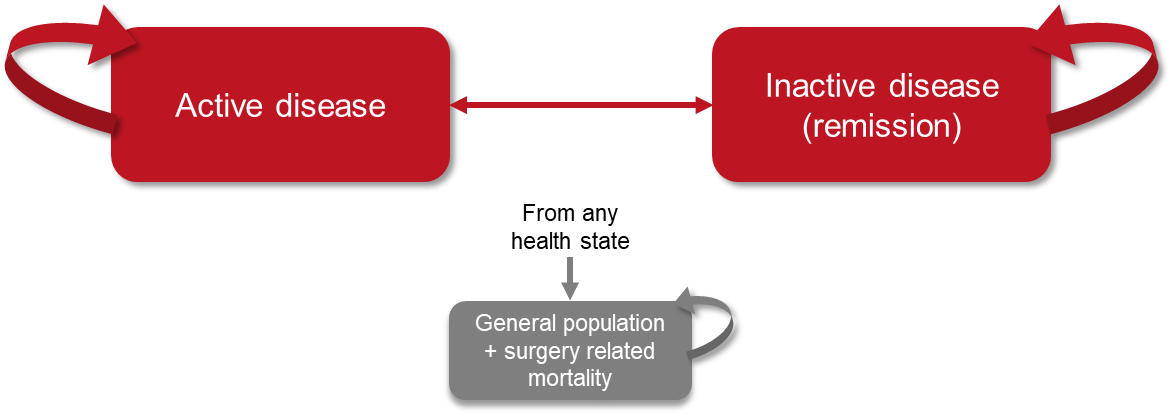


**Supplementary Figure 2. Proportion of patients on treatment in A) “Top-down” and B) “Accelerated step-up”.**

**A**


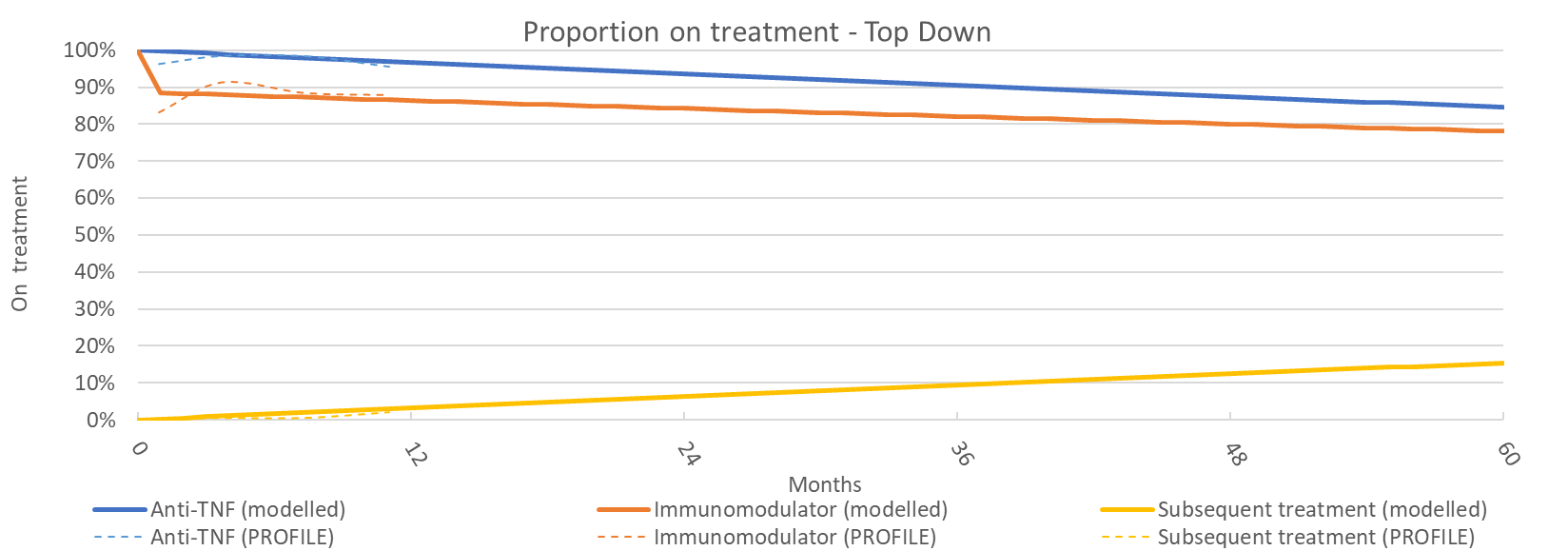


**B**


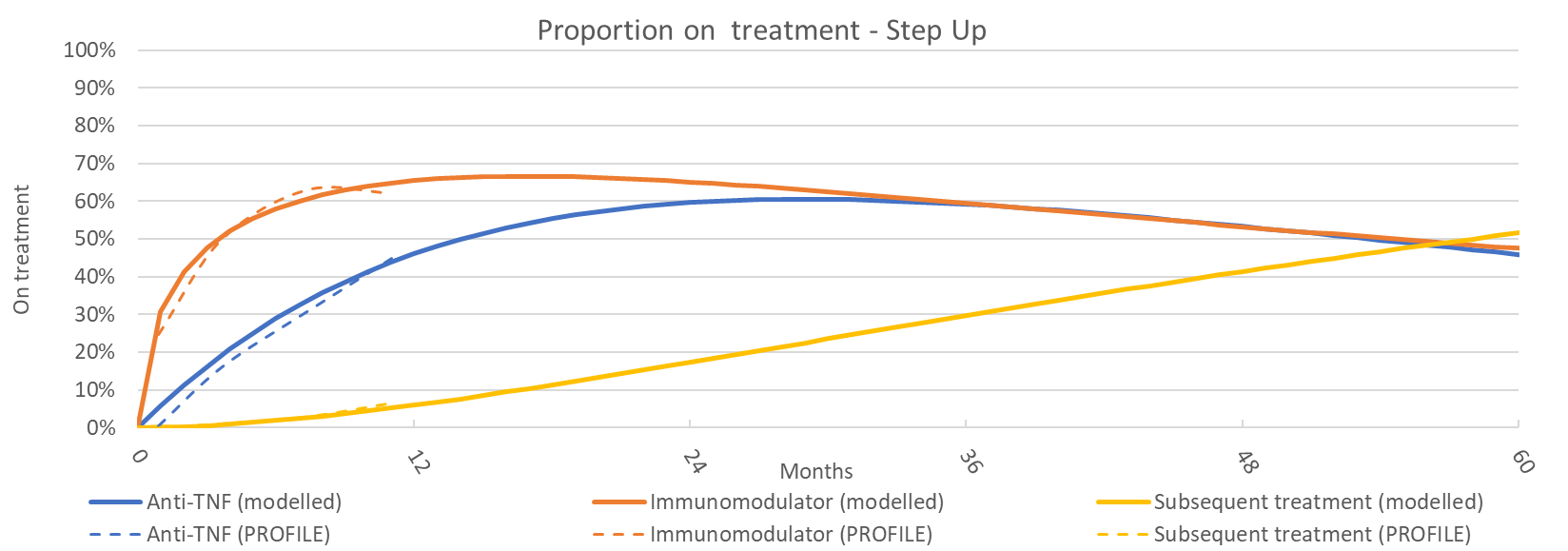


**(A)** Modelled (solid lines) and actual (dashed lines) proportions of patients on each treatment in the “top-down” arm. Data from PROFILE was used to estimate long-term time on treatment. **(B)** Actual data (dashed lines) from PROFILE for the proportions on each treatment in the “accelerated step-up” arm. The modelled curves (solid lines) also incorporated time on treatment data from the PANTS study,^21^ to estimate long-term time on treatment in the cost-effectiveness analysis model.

**Supplementary Figure 3. Base-case cost-effectiveness results for “top-down” versus “accelerated step-up” treatment assessing A) Total costs and B) QALYs.**

**A**

**
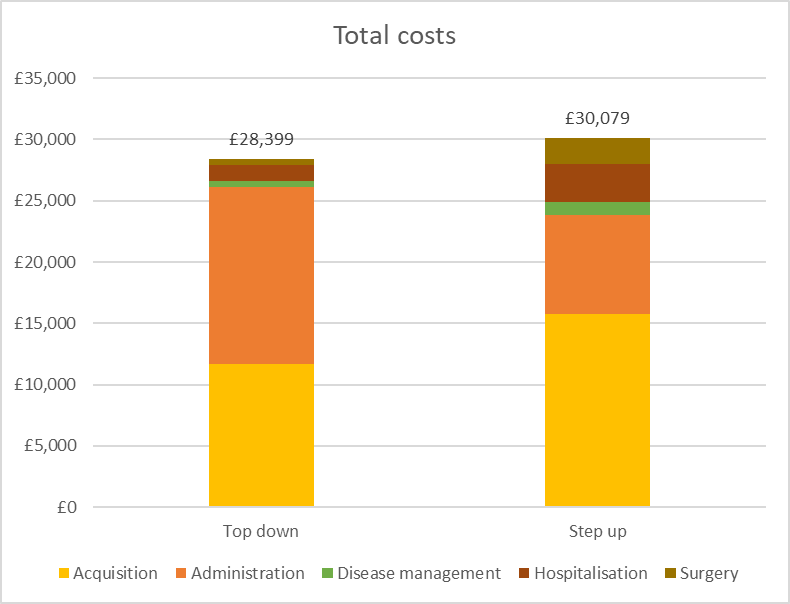
**

**B**


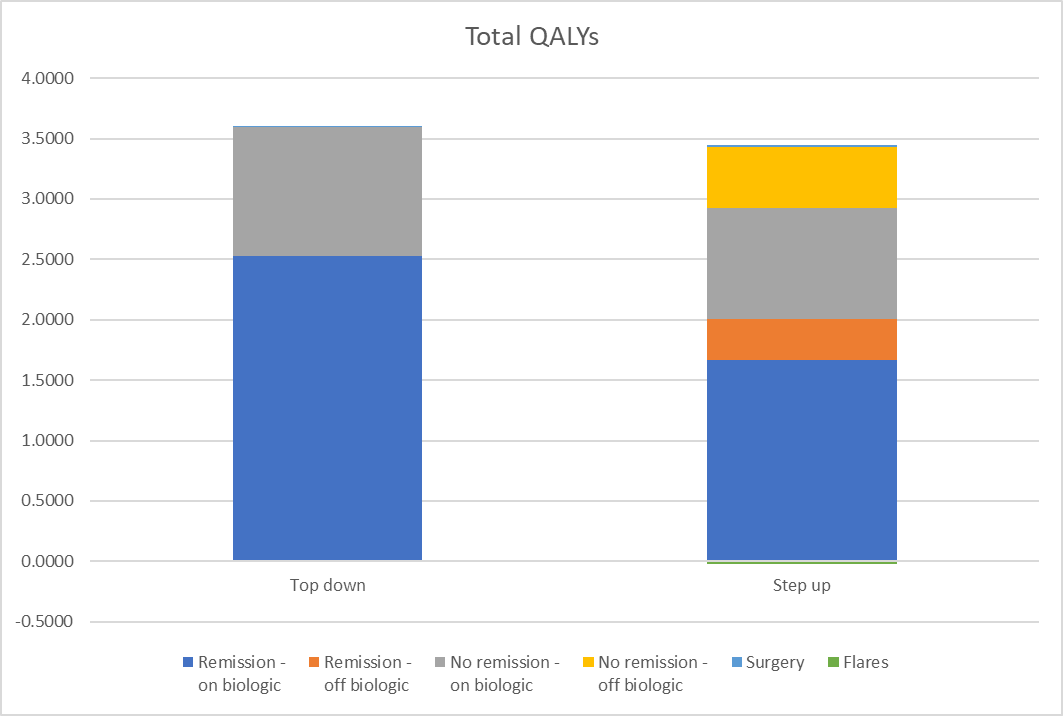


**Supplementary Figure 4. Cost-effectiveness acceptability curve, “top-down” versus “accelerated step-up”.**


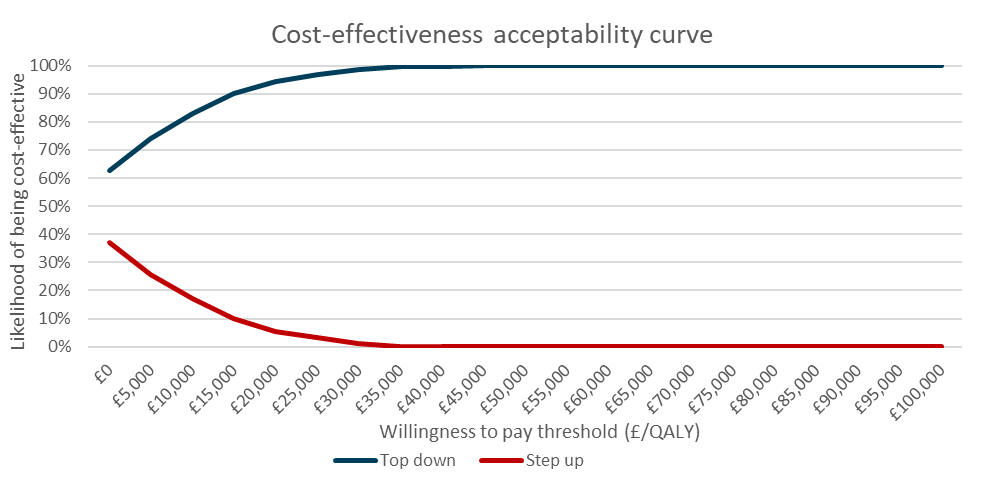


**CHEERS 2022 checklist.**

| **Section/topic** | **Item no.** | **Guidance for reporting** | **Reported in section** |
| --- | --- | --- | --- |
| **Title** | 1 | Identify the study as an economic evaluation and specify the interventions being compared. | __Title__ |
| **Abstract** | | |  |
| Abstract | 2 | Provide a structured summary that highlights context, key methods, results, and alternative analyses. | __Abstract__ |
| **Introduction** | | |  |
| Background and objectives | 3 | Give the context for the study, the study question, and its practical relevance for decision making in policy or practice. | __Introduction__ |
| **Methods** | | |  |
| Health economic analysis plan | 4 | Indicate whether a health economic analysis plan was developed and where available. | __ Not reported__ |
| Study population | 5 | Describe characteristics of the study population (such as age range, demographics, socioeconomic, or clinical characteristics). | __Results__ |
| Setting and location | 6 | Provide relevant contextual information that may influence findings. | __Methods – Study design and participants__ |
| Comparators | 7 | Describe the interventions or strategies being compared and why chosen. | __Methods – Study design and participants__ |
| Perspective | 8 | State the perspective(s) adopted by the study and why chosen. | __Methods - Model structure__ |
| Time horizon | 9 | State the time horizon for the study and why appropriate. | __Methods - Model structure__ |
| Discount rate | 10 | Report the discount rate(s) and reason chosen. | __Methods - Model structure__ |
| Selection of outcomes | 11 | Describe what outcomes were used as the measure(s) of benefit(s) and harm(s). | __Methods - Model structure__ |
| Measurement of outcomes | 12 | Describe how outcomes used to capture benefit(s) and harm(s) were measured. | __Method - Data__ |
| Valuation of outcomes | 13 | Describe the population and methods used to measure and value outcomes. | __Method - Transition probabilities__ |
| Measurement and valuation of resources and costs | 14 | Describe how costs were valued. | __Method – Costs__ |
| Currency, price date, and conversion | 15 | Report the dates of the estimated resource quantities and unit costs, plus the currency and year of conversion. | __Method – health resource use__ |
| Rationale and description of model | 16 | If modeling is used, describe in detail and why used. Report if the model is publicly available and where it can be accessed. | __Methods - model___ |
| Analytics and assumptions | 17 | Describe any methods for analysing or statistically transforming data, any extrapolation methods, and approaches for validating any model used. | __Supplementary appendix__ |
| Characterizing heterogeneity | 18 | Describe any methods used for estimating how the results of the study vary for subgroups. | __Not applicable__ |
| Characterizing distributional effects | 19 | Describe how impacts are distributed across different individuals or adjustments made to reflect priority populations. | __ Not applicable__ |
| Characterizing uncertainty | 20 | Describe methods to characterize any sources of uncertainty in the analysis. | __Method – base case analysis and sensitivity analysis__ |
| Approach to engagement with patients and others affected by the study | 21 | Describe any approaches to engage patients or service recipients, the general public, communities, or stakeholders (such as clinicians or payers) in the design of the study. | __Patient and public involvement section__ |
| **Results** | | |  |
| Study parameters | 22 | Report all analytic inputs (such as values, ranges, references) including uncertainty or distributional assumptions. | __Results__ |
| Summary of main results | 23 | Report the mean values for the main categories of costs and outcomes of interest and summarize them in the most appropriate overall measure. | __Results __ |
| Effect of uncertainty | 24 | Describe how uncertainty about analytic judgments, inputs, or projections affect findings. Report the effect of choice of discount rate and time horizon, if applicable. | __Results – Costs__ |
| Effect of engagement with patients and others affected by the study | 25 | Report on any difference patient/service recipient, general public, community, or stakeholder involvement made to the approach or findings of the study | __Acknowledgements __ |
| **Discussion** | | |  |
| Study findings, limitations, generalizability, and current knowledge | 26 | Report key findings, limitations, ethical or equity considerations not captured, and how these could affect patients, policy, or practice. | __Discussion__ |
| **Other relevant information** | | | |
| Source of funding | 27 | Describe how the study was funded and any role of the funder in the identification, design, conduct, and reporting of the analysis | __ Funding__ |
| Conflicts of interest | 28 | Report authors conflicts of interest according to journal or International Committee of Medical Journal Editors requirements. | __Conflicts of interest__ |
